# Modulation of Amyloid‐beta Metabolism by TIMP2 and MMP2 Through Extracellular Matrix Interactions in Mouse Models of Alzheimer's Pathology

**DOI:** 10.1002/alz70855_101110

**Published:** 2025-12-23

**Authors:** Alejandro B. Grau‐Perales, Suhani Yerapathi, Ana Catarina Ferreira, Sarah M Philippi, Brittany M. Hemmer, Jacob L. Rosenstadt, Claudia De Sanctis, John F. Crary, Joseph M. Castellano

**Affiliations:** ^1^ Icahn School of Medicine at Mount Sinai, Ronald M. Loeb Center for Alzheimer's disease, Nash Family Department of Neuroscience, Friedman Brain Institute, New York, NY, USA; ^2^ Icahn School of Medicine at Mount Sinai, New York, NY, USA

## Abstract

**Background:**

Alzheimer's disease (AD) is characterized by several pathological processes, including amyloid‐beta (Aβ) accumulation and neuroinflammation, both of which have been linked to extracellular matrix (ECM) dyshomeostasis. We previously reported that the youth‐associated factor TIMP2 revitalizes brain function in the context of aging and regulates ECM accumulation through its essential role in matrix metalloproteinase‐2 (MMP2) activation, an enzyme predominantly released by reactive astrocytes in response to injury. How TIMP2 may regulate AD pathology through its roles in ECM remodeling remains unexplored.

**Method:**

We characterized TIMP2 expression using immunohistochemistry in human AD and mouse brain tissue. Mouse hippocampus and cortex from two mouse models of Aβ pathology in which TIMP2 has been targeted was assessed for Aβ pathology, astrogliosis, accumulation of ECM components, as well as MMP2 expression. The impact of ECM accumulation on Aβ metabolism was evaluated through intrahippocampal injections of Chondroitinase ABC (ChABC). We also investigated the impact of TIMP2 rescue on AD pathology and behavioral performance using a vrial‐mediated overexpression approach.

**Result:**

TIMP2 expression differs significantly in human AD tissue and amyloid‐expressing mice relative to their respective controls. Mice in which TIMP2 had been targeted exhibit exacerbated amyloid pathology and astrogliosis in several brain regions, with increased expression of MMP2 and CSPGs, indicative of altered ECM homeostasis. Despite showing increased astrocyte recruitment around plaques, mice lacking TIMP2 have larger, more numerous plaques and greater astrocyte‐to‐plaque distance. Further analyses revealed more CSPG‐Aβ overlap but less MMP2‐Aβ overlap in mice lacking TIMP2. APP processing remained unchanged between TIMP2 genotypes, suggesting impaired Aβ clearance when modulating TIMP2. Intrahippocampal targeting of the ECM appears to increase astrocyte recruitment around plaques in amyloid‐bearing mice with coincident reductions in plaque size and number and enhanced MMP2‐Aβ overlap. TIMP2 overexpression in aged mice exhibiting beta‐amyloidosis improves cognitive performance, decreases Aβ plaque load, and reduces ECM accumulation.

**Conclusion:**

TIMP2 deficiency exacerbates amyloid pathology by affecting MMP2‐associated astrocyte modulation of ECM. Restoring TIMP2 improves cognitive and pathological outcomes, highlighting its therapeutic potential in AD. These findings suggest that TIMP2‐targeted strategies may represent a novel approach for addressing amyloid accumulation and downstream effects in AD. Funded by: NHI/NIA 1RF1AG072300, R01AG061382, Alzheimer's Association 24AARF‐1201458

